# A dual role for AMP‐activated protein kinase (AMPK) during neonatal hypoxic–ischaemic brain injury in mice

**DOI:** 10.1111/jnc.13034

**Published:** 2015-02-24

**Authors:** Catherine I. Rousset, Fiona C. Leiper, Anton Kichev, Pierre Gressens, David Carling, Henrik Hagberg, Claire Thornton

**Affiliations:** ^1^ Division of Imaging Sciences and Biomedical EngineeringCentre for the Developing BrainKing's College LondonKing's Health PartnersSt. Thomas’ HospitalLondonUK; ^2^ Redox Metabolism GroupMRC Clinical Sciences CentreImperial CollegeLondonUK; ^3^ Inserm, U1141ParisFrance; ^4^ Université Paris DiderotSorbonne Paris CitéUMRS 1141ParisFrance; ^5^ Cellular Stress GroupMRC Clinical Sciences CentreImperial CollegeLondonUK; ^6^ Perinatal CentreInstitutes of Clinical Sciences & Physiology and NeuroscienceSahlgrenska AcademyUniversity of GothenburgGothenburgSweden

**Keywords:** AMPK, hypoxia, ischaemia, neonatal, oxygen–glucose deprivation

## Abstract

Perinatal hypoxic–ischaemic encephalopathy (HIE) occurs in 1–2 in every 1000 term infants and the devastating consequences range from cerebral palsy, epilepsy and neurological deficit to death. Cellular damage post insult occurs after a delay and is mediated by a secondary neural energy failure. AMP‐activated protein kinase (AMPK) is a sensor of cellular stress resulting from ATP depletion and/or calcium dysregulation, hallmarks of the neuronal cell death observed after HIE. AMPK activation has been implicated in the models of adult ischaemic injury but, as yet, there have been no studies defining its role in neonatal asphyxia. Here, we find that in an *in vivo* model of neonatal hypoxia–ischaemic and in oxygen/glucose deprivation in neurons, there is pathological activation of the calcium/calmodulin‐dependent protein kinase kinase β (CaMKKβ)‐AMPKα1 signalling pathway. Pharmacological inhibition of AMPK during the insult promotes neuronal survival but, conversely, inhibiting AMPK activity prior to the insult sensitizes neurons, exacerbating cell death. Our data have pathological relevance for neonatal HIE as prior sensitization such as exposure to bacterial infection (reported to reduce AMPK activity) produces a significant increase in injury.

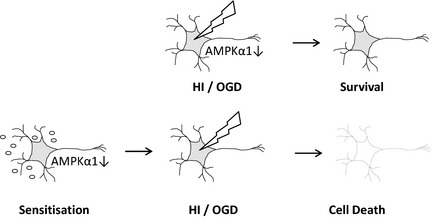

We show that in an *in vivo* model of neonatal hypoxia–ischaemic and in oxygen/glucose deprivation in neurons, there is a pathological activation of the CaMKKβ‐AMPKα1 signalling pathway. Inhibiting AMPK during OGD promotes neuronal survival; conversely, inhibiting AMPK prior to OGD exacerbates cell death. Our data have clinical relevance as prior sensitization (e.g. exposure to bacterial infection reducing AMPK activity) increases injury. AMPK, AMP‐activated protein kinase; HI, hypoxia–ischaemia; OGD, oxygen–glucose deprivation.

**Abbreviations used**
ACC: acetyl‐CoA carboxylase
AICAR: 5‐aminoimidazole‐4‐carboxamide 1‐β‐D‐ribofuranoside
AMPK: AMP‐activated protein kinase
CaMKKβ: calcium/calmodulin‐dependent protein kinase kinase β
DIV: days *in vitro*
HIE: hypoxic–ischaemic encephalopathy
HI: hypoxia–ischaemia
LDH: lactate dehydrogenase
LPS: lipopolysaccharide
MAP2: microtubule‐associated protein 2
MCAO: middle cerebral artery occlusion
MOPS: 3‐(*N*‐morpholino)propanesulfonic acid
OGD: oxygen–glucose deprivation
PVDF: polyvinylidene fluoride

Moderate‐to‐severe hypoxic–ischaemic encephalopathy (HIE), caused by a lack of oxygen or blood flow to the brain around the time of birth, affects 1–2 babies in every 1000 in the UK and far more in the developing world (Kurinczuk *et al*. 2010). The consequences for infants and parents affected by HIE are devastating; 15–20% will die in the post‐natal period and a further 25% will develop severe and long‐lasting neurological impairments. Currently, therapeutic hypothermia is the only available treatment and, although not successful in all cases, will double the chance of normal survival (Azzopardi *et al*. 2014). However, the success of therapeutic hypothermia provides proof‐of‐concept that intervention post injury can be effective.

It is now well accepted that although there is some brain damage during HIE, the majority of cell death occurs after a delay as a result of a secondary neural energy failure (Azzopardi *et al*. 1989; Lorek *et al*. 1994; Blumberg *et al*. 1997; Gilland *et al*. 1998). AMP‐activated protein kinase (AMPK) is a sensor of cellular stress, activated by phosphorylation by one of two upstream kinases, Liver Kinase B1 (LKB1) or calcium/calmodulin‐dependent protein kinase kinase β (CaMKKβ), in response to depletion of ATP or alterations in intracellular calcium concentrations (Carling *et al*. 2008; Steinberg and Kemp 2009). AMPK is a heterotrimer consisting of a catalytic (α1 or α2) subunit, coupled to two regulatory subunits (β1 or β2; and γ1, γ2 or γ3). Maximal activation of AMPK is achieved by phosphorylation at threonine 172 within the α subunit, allosteric activation by AMP and inhibition of dephosphorylation by ADP at nucleotide‐binding sites on the γ subunit (Xiao *et al*. 2007, 2011). Once activated, AMPK shuts down ATP consuming, anabolic pathways and promotes ATP‐generating, catabolic pathways (Steinberg and Kemp 2009). As such, many metabolic substrates have been identified including regulators of fatty acid and cholesterol synthesis, mitochondrial biogenesis and protein synthesis. Therefore, AMPK has become a potential therapeutic target because of its role in diseases which have perturbation in energy homeostasis such as type 2 diabetes, cancer and inflammation (Carling *et al*. 2012; Hardie 2013).

Compared with peripheral tissues, the role of AMPK in the brain is still being delineated. Neurons have small energy stores but high energy demands and activation of AMPK can occur in response to a number of pathologically relevant stresses such as glucose deprivation (Culmsee *et al*. 2001), hypoxia (Gusarova *et al*. 2011; Mungai *et al*. 2011) and excitotoxicity (Weisova *et al*. 2009; Concannon *et al*. 2010). Increasingly, AMPK activation is implicated in the development of neurological disorders (Ju *et al*. 2011; Vingtdeux *et al*. 2011; Lim *et al*. 2012; Mochel *et al*. 2012), although as with the neuronal studies, it is unclear whether this activation is beneficial (Chen *et al*. 2009; Choi *et al*. 2013) or deleterious (McCullough *et al*. 2005; Li *et al*. 2007; Vingtdeux *et al*. 2010).

The majority of studies of AMPK in the mechanism of hypoxic–ischaemic injury have utilized adult middle cerebral artery occlusion (MCAO) models of stroke (Li *et al*. 2010; Choi *et al*. 2013; Jin *et al*. 2014; Venna *et al*. 2014). However, it is increasingly clear that such results cannot be easily translated into the neonatal brain environment and many mechanisms of cell death are different in the immature versus the adult brain (Ferriero 2004; Vannucci and Hagberg 2004; Wang *et al*. 2009, 2010; Hagberg *et al*. 2014). It therefore becomes very relevant to investigate specific mechanisms in appropriate neonatal models.

In this study, we examine the contribution of AMPK activation to the development of hypoxic–ischaemic brain injury in neonatal mouse brain. We find that AMPK activity is up‐regulated during the injury but quickly returns to baseline levels on removal of the insult. In an *in vitro* model of hypoxia–ischaemia (HI) in primary neurons, activation of AMPK during the injury results in increased cell death, whereas inhibition of AMPK during the insult protects the cells, implicating AMPK in the injury process. Conversely, we find that inhibition of AMPK *prior* to injury sensitizes neurons to subsequent insult. Taken together, these data show a dual role of AMPK in HI brain injury in neonates depending on whether it is modulated *prior* or *during* the insult.

## Materials and methods

### Materials

Unless otherwise stated, all reagents were purchased from Sigma‐Aldrich (Dorset, UK) with cell culture medium and supplements from Life Technologies (Paisley, UK).

### *In vivo* model of HI

Experiments were conducted in Sweden under the guidance of the Swedish Ethical Authorization of Gothenburg University (Jordbruksverket, Jönköping, Sweden). Animals were bred in‐house and pups of both sexes used for the injury model. Post‐natal day (P)9 C57/Bl6 mice were subjected to left common carotid artery ligation followed by 50 min of hypoxia in a 10% oxygen environment stabilized at 36°C (Rice *et al*. 1981; Vannucci and Vannucci 1997; Hedtjarn *et al*. 2002). At various time points during and after the insult, animals were quickly decapitated and the head snap frozen in liquid nitrogen to avoid artifactual activation of AMPK. Brains were dissected in a cryostat (−20°C) to isolate and harvest hippocampus, cortex and striatum. For brain damage assessment, animals were killed by overdose of anaesthetic (pentobarbital) and perfused with saline (Baxter, Newbury, UK) for 5 min followed by a perfusion fixation with Histofix^®^. After various dehydration steps, brains were paraffin embedded and cut in coronal section of 6 μm. Brain damage was assessed by microtubule‐associated protein 2 immunohistochemistry of the ipsi‐ and contralateral hemispheres on coronal sections as described previously (Jarlestedt *et al*. 2013).

### Preparation of primary neurons

Animal use was in accordance with local rules (King's College, London) and with the regulations and guidance issued under the Animals (Scientific Procedures) Act (1986). CaMKKβ‐deficient primary neurons were prepared from animals kindly donated by Prof K. P. Giese (KCL) and wild‐type neurons were prepared from time‐mated dams purchased from Charles River Laboratories Inc., Harlow, UK. Pregnant C57/Bl6 mice were killed by schedule 1 at 13.5–14.5 days of gestation and embryonic cortical neurons prepared as described previously (Thornton *et al*. 2011). Cells were plated at a density of 2 × 10^6^ cells/6 cm plate and propagated for a minimum of 9 days *in vitro* (DIV) before treatments were applied. Arabinocytidine (5 μM) was added once at DIV 2–4 to prevent propagation of dividing cells and ensure the purity of the neuronal culture.

### *In vitro* oxygen–glucose deprivation

At DIV 10–12, neurons were subjected to oxygen–glucose deprivation (OGD) in neurobasal‐A medium lacking glucose and incubated in a hypoxia chamber (Billups‐Rothenburg Inc. Del Mar, CA, USA) filled with an anoxic atmosphere of 5% CO_2_ balanced in nitrogen at 37°C for 2 h. Cells were incubated with 5‐aminoimidazole‐4‐carboxamide 1‐β‐d‐ribofuranoside a precursor for ZMP, an AMP mimetic (AICAR; 2 mM), the calcium ionophore ionomycin (an activator of CaMKKβ; 2 μM final concentration), or the CaMKKβ inhibitor STO609 (10 μg/mL), all from Tocris Bioscience, Bristol, UK. Pharmacological modulators were incubated with the cells either during OGD (OGD experiments), or for 2 h at 3 h or 24 h prior to OGD (pre‐treatment experiment). Culture medium was assayed for release of lactate dehydrogenase (LDH) (described below), and cell lysates prepared in ice‐cold HEPES Buffer A (50 mM HEPES (pH 7.5), 50 mM sodium fluoride, 5 mM sodium pyrophosphate, 1 mM EDTA, +1 × protease inhibitors) containing 1% (v/v) Triton X‐100.

### Caspase 3/7 activity measurements

Primary neurons were subjected to OGD and measurement of active caspases (3/7) post insult determined at time points from 0 to 30 h using the CellEvent Caspase 3/7 Green peptide (2 μM final concentration, Life Technologies) in accordance with manufacturer's instructions. Hoechst stain (2 μg/mL) was also included in the media. After incubation, cells were washed and fluorescence determined at 530 nm (caspase 3/7) and 460 nm (Hoechst) on a platereader (GloMax Multi+; Promega, Southampton, UK.). Background fluorescence measurements were removed and the ratio of caspase fluorescence per cell number calculated. Data were normalized to a non‐OGD control and compared with the 0‐h time point.

### LDH assay

Cell death was assessed using an LDH cytotoxicity kit (Sigma, Dorset, UK) in accordance with manufacturer's instructions. LDH released into the medium was used to measure cell membrane permeability and therefore act as a surrogate for cell death. LDH concentrations were determined by absorbance at 450 nm (Glomax; Promega) and normalized for background absorbance at 750 nm and medium‐alone controls.

### AMPK activity

Immunoprecipitation of AMPK subunits and assay of activity by incorporation of radiolabelled MgATP into a substrate peptide has been described previously (Woods *et al*. 1996; Thornton *et al*. 2011).

### Western blots

Proteins were resolved on 10–12% (w/v) NuPAGE BisTris gels in 3‐(*N*‐morpholino)propanesulfonic acid buffer (Life Technologies) and transferred to polyvinylidene fluoride membrane. Proteins were analysed by western blot using the following antibodies: phospho‐acetyl‐CoA carboxylase, AMPKα (pT172) and AMPKβ (all at 1 : 1000; Cell Signaling Technology, Beverly, MA, USA).

### Analysis of mRNA by qRT‐PCR

For preparation of total RNA, primary neurons were harvested in TRIzol (1 mL per 1 × 10^6^ cells; Life Technologies), and total RNA was extracted in chloroform as per manufacturer's instructions. The resulting RNA was purified using an RNeasy column (Qiagen, Manchester, UK) and eluted in DNase/RNase‐free dH_2_O. Total RNA (200 ng) was analysed using Taqman gene expression assays and RNA‐to‐C_T_ kit (Life Technologies) on a StepOneplus Cycler (Life Technologies). Data were normalized to the expression of Glyceraldehyde 3‐phosphate dehydrogenase (GAPDH) and to controls using the delta‐delta C_T_ method (Livak and Schmittgen 2001). Primer pairs used in the study are as follows: GAPDH (Mm99999915), AMPKα1 (*Prkaa1*, Mm01296700), AMPKα2 (*Prkaa2*, Mm01264789) from Life Technologies.

### Statistics

Results are expressed as means ± SEM from three or more independent experiments. Statistical analysis was performed using Prism (GraphPad Software Inc., San Diego, CA, USA). Statistical significance between two conditions was established with a two‐tailed Student's *t*‐test, whereas significance among multiple datasets was determined by anova, followed by *post‐hoc* Bonferroni multiple comparison tests. Results were considered significant at *p* < 0.05.

## Results

### AMPK is activated early in development

**Figure 1:**
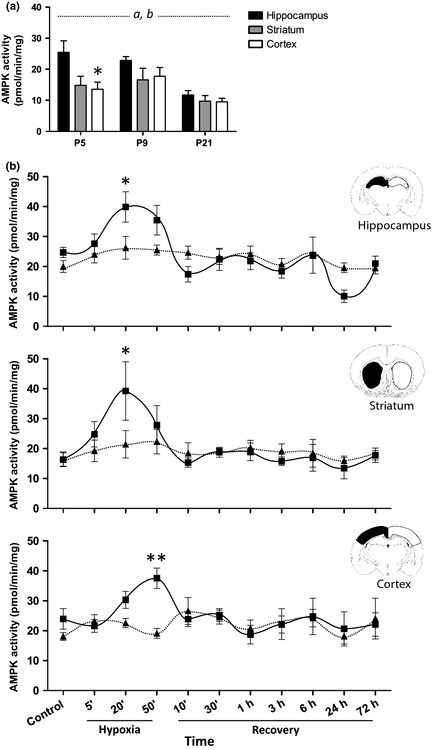
AMPK is activated during hypoxic–ischaemic injury *in vivo*. (a) Protein samples from hippocampus (black bars), striatum (grey bars) and cortex (white bars) were generated from snap‐frozen, uninjured mouse brains at the times indicated and AMPK activity measured. Significant differences were observed with respect to brain region (*a*,* p* = 0.0031) and age of animal (*b*,* p* = 0.0001). *Post hoc* tests revealed that AMPK activity was initially significantly higher in the hippocampus (*p < 0.05) than the cortex but activity in all three regions declined as the animal aged (*n* = 6–7). (b) Brain samples from sham‐injured (triangles) and hypoxia–ischaemia (HI)‐injured (squares) mice were taken at times indicated either during the insult itself or during the recovery phase. Protein lysates from the hippocampus (top graph), striatum (middle graph) and cortex (bottom graph) were prepared and AMPK assayed as above. AMPK is activated *in vivo* during HI (*p < 0.05, **p < 0.01) but returns to baseline during recovery (*n* = 6–7).

To establish a baseline of activation for AMPK in our animals, we assessed activity at different developmental stages from P5 to P21. Our data show that AMPK activation is not homogeneous within the brain during development. Both brain region and time had a significant effect on the variation in AMPK activity and in addition, *post‐hoc* analysis revealed a significant difference in AMPK activity between hippocampus and cortex at P5 (Fig. 1a). However, as previously suggested (Saito *et al*., 2009), we found that there was an overall decrease in AMPK activity in the mouse brain during this period (Fig. 1a).

### AMPK is activated *in vivo* in response to hypoxic–ischaemic insult

To test our hypothesis that, considering the large energy failures associated with HI, AMPK should be activated, we measured AMPK activity on HI injured and uninjured mice. We subjected P9 mice to HI insult using the well‐characterized Vannucci model (Rice *et al*. 1981; Vannucci and Vannucci 1997); the insult generated approximately 25% tissue loss localized to the hippocampus, cortex and striatum (Figure S1). As expected, we observed a rapid increase in AMPK activity in these three ipsilateral areas during the HI insult compared with equivalent contralateral regions although the maximal kinase activity occurred at slightly different times in the different regions. Remarkably, this activation quickly returned to baseline levels once the animals were returned to normoxia (Fig. 1b). These data provide *in vivo* confirmation that up‐regulation of AMPK activity is involved in neonatal HI.

### AMPK is activated in primary neurons in response to oxygen–glucose deprivation

**Figure 2:**
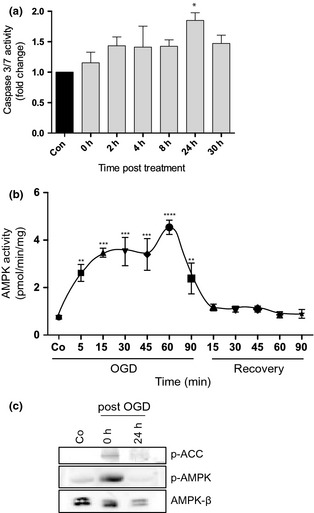
AMPK is activated in primary neurons during oxygen–glucose deprivation (OGD). Primary neurons were subjected to OGD for 90 min followed by a return to normoxia and complete medium. (a) Primary neurons were incubated in the presence of a fluorescent caspase 3 activity probe. Fluorescence was measured at the time points indicated, normalized to number of cells and expressed as fold change compared with a non‐OGD control. Caspase activity at 24 h was significantly greater than that measured at 0 h (*n* = 3; *p < 0.05). (b) Protein lysates were prepared at time points during the insult and subsequent recovery and assayed for AMPK activity. AMPK activity rapidly increased, peaking at 60 min before returning to baseline during recovery (**p < 0.01, ***p < 0.001, ****p < 0.0001; *n* = 4). (c) Protein lysates prepared at 0 and 24 h post insult were analysed by western blot for active AMPK (p‐AMPK) and phosphorylated acetyl‐CoA carboxylase (p‐ACC), an AMPK substrate. Blots were controlled for total AMPK expression using an antibody recognizing the ubiquitous AMPK‐β regulatory subunits (AMPK‐β; *n* = 3). Phosphorylation of ACC confirms up‐regulation of functional AMPK activity during the insult.

To further investigate the role of AMPK during HI, we generated primary cortical neurons from E14.5 mice and subjected them to an *in vitro* OGD insult for 90 min. The activation of cell death mechanisms was confirmed by the appearance of active caspase 3 which began at 2 h post insult and was sustained for at least 24 h (Fig. 2a). A dissection of the timeline of AMPK activation during and after the insult revealed that AMPK activity increased rapidly during the insult, reaching a peak at 60 min (Fig. 2b). Interestingly, the activation died away rapidly post insult when the cells were returned to normoxia and complete medium (Fig. 2b) correlating with our *in vivo* observations. As might be expected, AMPK activation resulted in phosphorylation of acetyl‐CoA carboxylase, a well‐characterized substrate of AMPK (Fig. 2c).

### AMPK activation during OGD is mediated by the CaMKKβ‐AMPKα1 pathway

**Figure 3:**
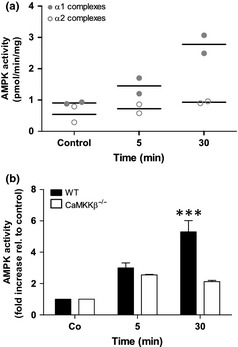
AMPK activation during oxygen–glucose deprivation (OGD) is mediated by the CaMKKβ‐AMPKα1 pathway. Wild‐type (WT) and/or CaMKKβ‐deficient (CaMKKβ^−/−^) primary neurons were subjected to OGD for 5 min or 30 min and protein lysates prepared. (a) AMPKα1‐ (closed circles) or AMPKα2‐specific immune complexes (open circles) were isolated from OGD‐treated WT neurons and assayed for activity compared with untreated controls. There was a striking difference observed in that the majority of AMPK activity was attributed to AMPKα1‐containing complexes (*n* = 2). (b) AMPK activity was assessed in WT and CaMKKβ‐deficient neurons subjected to OGD. The absence of CaMKKβ prevented full AMPK activation at 30 min (***p < 0.001; *n* = 3–6).

Previous studies suggested that in an adult (MCAO) stroke model infarct volume was determined by the activation of AMPKα2 complexes (Li *et al*. 2007, 2010). However, more recently, it has been suggested that hypoxic stress acts through calcium influx, triggering CaMKKβ‐mediated AMPKα1 activation (Gusarova *et al*. 2011; Mungai *et al*. 2011). We therefore isolated AMPKα1‐ or AMPKα2‐specific immune complexes from neuronal lysates prepared at various time points during OGD and found that the changes in AMPK activity were largely mediated by AMPKα1 (Fig. 3a). Moreover, analysis of OGD lysates from WT and CaMKKβ‐deficient primary neurons found that although there was increases in AMPK activity in both lysates at 5 min (presumably because of depletion of ATP), this was not maintained in the CaMKKβ‐deficient neurons at 30 min (Fig. 3b). This suggests that in our model, OGD up‐regulates AMPKα1 complex activity via activation of CaMKKβ.

### Prolonged activation of AMPK is detrimental in the neuronal response to oxygen–glucose deprivation

**Figure 4:**
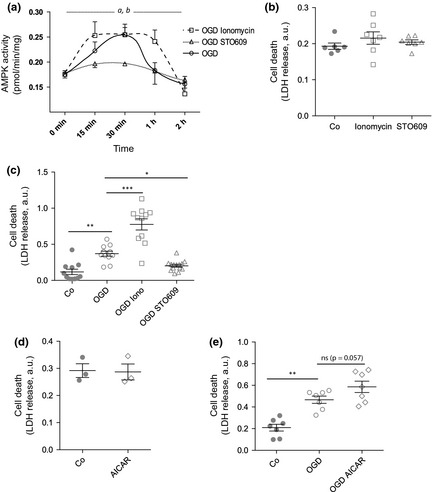
Prolonged activation of AMPK during oxygen–glucose deprivation (OGD) increases cell death. Primary neurons were subjected to OGD for 2 h either alone or in the presence of the calcium/calmodulin‐dependent protein kinase kinase β (CaMKKβ) activator ionomycin (2 μM; OGD Iono) or the CaMKK inhibitor STO609 (10 μg/mL; OGD STO). (a) Protein lysates were generated at the times shown and assayed for AMPK activity. Significant differences were observed with respect to treatment (*a*,* p* = 0.032) and time (*b*,* p* < 0.0001). The presence of STO609 (open triangles) during the insult prevented the activation of AMPK, whereas the presence of ionomycin (open squares) increased the rate of activation of AMPK and its duration. (b) Primary neurons were incubated for 2 h in the presence of DMSO vehicle (Co; closed circles), ionomycin (2 μM; open squares) or STO609 (10 μg/mL; open triangles) and cell death measured (using release of lactate dehydrogenase (LDH) into the medium as a surrogate). The LDH measurements shown represent normalized absorbance at 450 nm. No significant differences were observed between the groups (*n* = 6–7) (c). OGD resulted in a significant increase in LDH production (open circles) compared with control (closed circles), which was further exacerbated in the presence of ionomycin (open squares). Conversely, the presence of STO609 (open triangles) significantly decreased the deleterious effect of the insult on neurons (*p < 0.05, **p < 0.01, ***p < 0.001; *n* = 11–12) (d). Primary neurons were incubated for 2 h in the presence of 5‐aminoimidazole‐4‐carboxamide 1‐β‐d‐ribofuranoside (AICAR) and cell death assessed as previously. No significant differences were observed between vehicle‐ and AICAR‐treated cells (*n* = 3). (e) As stated earlier, OGD resulted in a significant increase in LDH production (open circles) compared with control (closed circles). In line with the previous observation with ionomycin, AICAR‐treated neurons appeared to produce more LDH, but this did not reach significance (*n* = 7, **p < 0.01).

A number of pharmacological agents are capable of modifying AMPK activity. As our results implicated a CaMKKβ‐AMPKα1‐dependent pathway, we used our previously characterized system of ionomycin (activator)/STO609 (inhibitor) treatment to modulate AMPK activity in a CaMKKβ‐dependent manner (Thornton *et al*. 2008). We exposed neurons to OGD in the presence of ionomycin or STO609 to determine the effect of extreme activation or inhibition of AMPK on cell survival. As expected, STO609 significantly reduced AMPK activity during OGD (Fig. 4a). Conversely, AMPK appeared to be maximally activated by OGD alone, as no further increase in the magnitude of activation was observed on addition of ionomycin. However, OGD in the presence of ionomycin resulted in a more rapid (15 min) and sustained (> 1 h) AMPK activation (Fig. 4a). To determine the effect on cell survival, we incubated primary neurons with ionomycin or STO609 and measured release of LDH into the medium as a surrogate for cell death. Neither ionomycin nor STO609 alone altered neuronal survival (Fig. 4b); however, in combination, the prolonged activation of AMPK by ionomycin during OGD significantly increased cell death after the insult, whereas STO609 inhibition of AMPK had the opposite effect, increasing cell survival (Fig. 4c). To confirm that it was indeed the activation of AMPK and not just calcium dysfunction causing the changes in neuronal survival, we repeated the experiment using a calcium‐independent, classical activator of AMPK, AICAR, a precursor for ZMP, an AMP mimetic. AICAR treatment did not alter cell survival alone (Fig. 4d); however, although not significant, there was a trend towards increased LDH release, in a similar manner to that evoked by ionomycin (Fig. 4e). These data suggest that acute AMPK activation in response to OGD is detrimental to the survival of the neurons and suggests that targeting AMPK activation in the acute phase of injury may be beneficial.

### Decreasing AMPK prior to injury sensitizes neurons to OGD

**Figure 5:**
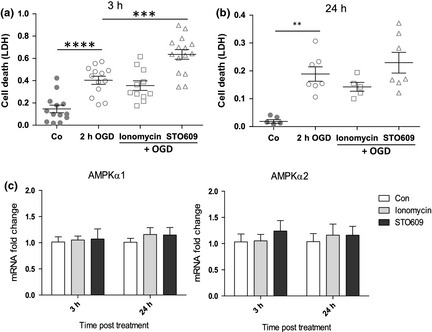
Pharmacological inhibition of AMPK prior to oxygen–glucose deprivation (OGD) sensitizes neurons to subsequent insult. (a) Primary neurons were incubated in the presence of vehicle, ionomycin or STO609, allowed to recover for 3 h in complete medium and then subjected to OGD. A significant increase in lactate dehydrogenase (LDH) release suggesting cell death post insult was observed in cultures which had been treated with STO609 (open triangles) compared with OGD alone (open circles). There was a slight protective effect of ionomycin (open squares) but this did not reach significance (***p < 0.001, ****p < 0.0001; *n* = 11–15). (b) The experiment outlined in (a) was repeated leaving an interval of 24 h between pre‐treatment and OGD. However, although the trend was the same, the effects of ionomycin and STO609 were not significant. (c) Primary neurons were treated with ionomycin and STO609 as previously and allowed to recover for 3 or 24 h. RNA was prepared from the cells and the expression of AMPKα1 (left panel) and AMPKα2 (right panel) determined by qRT‐PCR. There was no significant difference in AMPK mRNA expression observed between treated samples and control at either 3 or 24 h (*n* = 4).

Classical preconditioning is a paradigm in which a sublethal stress (e.g. mild OGD) is evoked prior to the onset of lethal injury (e.g. severe OGD). This sublethal exposure renders cells more resistance to injury and has been used successfully in neonatal rat models of HI (Gidday *et al*. 1994; Gustavsson *et al*. 2005). As the previous experiment demonstrated that sustained AMPK activity during OGD was detrimental, we sought to examine if pre‐treatment using AMPK modulators would protect the neurons from OGD. We exposed primary neurons to ionomycin or STO609 for 2 h and the cells were then allowed a 3‐h recovery before exposure to OGD. Cell survival was measured immediately after the insult and revealed that in neurons pre‐treated with STO609, OGD‐induced cell death was greatly exacerbated (Fig. 5a). Surprisingly, pre‐treatment with ionomycin did not significantly alter the cells’ response to OGD, although there seemed to be a trend towards protection (Fig. 5a). As the preincubation may take time to exert its effects, we repeated the experiment with a 24 h recovery time between the exposure and OGD. We observed a similar pattern as for 3 h; however, any changes in cell survival did not reach significance (Fig. 5b). One possibility for the altered behaviour of STO609 may be that as a consequence of prolonged inhibition, AMPK expression is up‐regulated in compensation and from our previous results, increased AMPK during OGD is deleterious (Fig. 4c). To rule out such homeostatic responses, we analysed the mRNA expression of AMPKα1 and AMPKα2 at 3 h and 24 h post treatment with AMPK modulators. We found that although both subunits were expressed at similar levels in neurons (Figure S2), there were no alterations in expression of either subunit in response to treatment or recovery time (Fig. 5c). Taken together, these data imply that inhibition of AMPK just prior to insult may sensitize neurons to HI injury, potentially through down‐regulation of AMPK activation‐mediated metabolic pathways.

## Discussion

AMPK is known for its role as energy sensor of the cell, working to restore energy balance by inhibiting energy‐consuming pathways and promoting energy‐producing pathways. It is now well recognized that HIE is associated with energy failure which correlates with delayed onset of injury. Our aim was to investigate the role of AMPK in the development of injury *in vivo* in HI models and *in vitro* in OGD.

Our results show that AMPK is rapidly activated both *in vivo* and *in vitro*. Prolonging the activation of AMPK during the insult results in increased cell death, suggesting that AMPK may contribute to the development of injury. This is borne out by the opposite experiment in which inhibition of AMPK activity during the insult confers significant protection. In non‐neuronal tissue, activation of AMPK is metabolically advantageous, preserving and restoring ATP levels, regulating glucose uptake, promoting autophagy and mitochondrial biogenesis (Ruderman *et al*. 2013). However, there is still controversy surrounding prolonged AMPK activity in the brain. AMPK promotes whole body energy homeostasis balancing hormone peptide signalling in the hypothalamus (Xue and Kahn 2006). In addition, activation of AMPK modulates excitotoxic glutamatergic signalling (Culmsee *et al*. 2001; Kuramoto *et al*. 2007). Longer term, potentially pathological activation of AMPK may occur during neurodegenerative disorders (Alzheimer's disease, Huntington's disease) in which there is an early but prolonged perturbation of energy metabolism (Chou *et al*. 2005; Vingtdeux *et al*. 2011; Lim *et al*. 2012). At a cellular level, elegant studies in primary cerebellar granule neurons identified a role for AMPK in response to transient glutamate excitation. This excitation resulted in a glucose‐dependent hyperpolarization of the mitochondrial membrane potential and activation of AMPK, facilitating the membrane localization of glucose transporter GLUT3 and providing neuronal tolerance (Weisova *et al*. 2012). This mechanism seems to underlie the protective properties of the Alzheimer's and Huntington's disease drug Laterpirdine/Dimebon, which can also activate AMPK, promote GLUT3 translocation and protect neurons from glutamate excitotoxicity (Weisova *et al*. 2013). However, the same group also suggests that prolonged activation of AMPK in neurons in response to a variety of stimuli is reported to up‐regulate the expression of pro‐apoptotic Bim leading to cell death (Concannon *et al*. 2010). Our results are consistent with this latter view in that prolonged AMPK activation was detrimental to cell survival when combined with injury and we also see an increase in apoptosis in primary neurons.

In rodent MCAO models of adult stroke, genetic ablation of AMPKα2 reduces infarct volume. In contrast, we find that it is AMPKα1 complexes that are critical in our insult model. However, there are a growing number of studies suggesting differential roles for AMPKα1 and AMPKα2, with AMPKα1 pathways more consistently linked with alteration in intracellular calcium and therefore modulated by CaMKKβ (Stahmann *et al*. 2006; Tamas *et al*. 2006; Thornton *et al*. 2011). Recently, two independent studies identified a hypoxia‐mediated CaMKKβ‐AMPKα1 pathway triggered in response to calcium release‐activated calcium channels (Gusarova *et al*. 2011; Mungai *et al*. 2011). Interestingly, this mechanism was pathological in nature as AMPKα1 activation resulted in down‐regulation of Na,K‐ATPase and subsequent lung dysfunction (Gusarova *et al*. 2011).

Several studies recently reported a protective role for AMPK during preconditioning. Ischaemic preconditioning was induced in adult rats by transient MCAO which up‐regulated AMPK activity and promoted autophagy. These animals showed reduced infarct volume and improved neurological function after a subsequent permanent MCAO compared with rats in which AMPK or autophagy had been inhibited (Jiang *et al*. 2014b). The same group showed that this beneficial preconditioning could also be achieved by a single dose of the AMPK activator, Metformin (Jiang *et al*. 2014a). However, this contradicts previous studies of brief MCAO preconditioning where it was the significant inhibition of AMPK activity which provided protection (Venna *et al*. 2012). In addition, preconditioning experiments in primary neurons using the AMPK activator AICAR found that the activation of AMPK was protective in a paradigm of NMDA‐induced excitotoxicity (Anilkumar *et al*. 2013). Although our experiments targeted the Ca^2+^‐ rather than the AMP/ADP‐mediated activation of AMPK, our findings are also consistent with a protective role for AMPK activity in preconditioning. Depletion of AMPK prior to insult significantly increased cell death, suggesting that the cells were sensitized to the injury. Analysis of AMPK mRNA levels after a recovery period suggested that they were unaffected by prolonged pre‐treatment with STO609 or ionomycin. It is interesting to speculate therefore that it is the metabolic maintenance role which AMPK performs in continuously balancing the physiological energy demands of the cell, which is critical.

**Figure 6:**
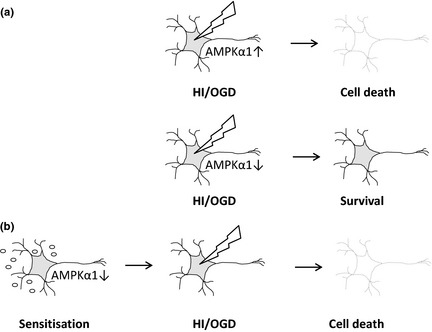
AMPK activation offers a dual target in the prevention of neuronal cell death. Based on our data, we hypothesize that prolonged activation of AMPK in neurons and animal models of hypoxia–ischaemia (HI) is deleterious suggesting that pharmacological inhibition of AMPK activity during the acute injury would be beneficial (a). Conversely, exposure of the neuron to pathological inhibitors of AMPK, as might occur in the presence of a bacterial infection, would render it more sensitive to a subsequent insult (b).

In contrast with the many physiological and pathological activators of AMPK, there are very few cases in which AMPK activity is inhibited *in vivo* and therefore sensitizing neurons by AMPK depletion may represent the first stage in a unique pathological cascade. One published inhibitor of AMPK activity *in vitro* and *in vivo* is the endotoxin lipopolysaccharide (LPS) derived from *E. coli* (Sag *et al*. 2008; Yang *et al*. 2010; Zhang *et al*. 2010). In addition, infection contributes to neuronal sensitization in response to neonatal hypoxic–ischaemic injury (Eklind *et al*. 2001, 2005; Hagberg and Mallard 2005). Interestingly, although it has been suggested that LPS‐mediated inhibition of AMPK mainly occurs in macrophages rather than neurons, it is the AMPKα1 complexes which are most affected (Sag *et al*. 2008; Yang *et al*. 2010). It is therefore tempting to speculate that infection‐mediated inhibition of AMPK sensitizes neurons and other neural cells to HI‐mediated injury (Fig. 6) particularly as recent studies suggest that neurons may possess the necessary components to sense bacterial infection, thus acting as a trigger for innate immunity signalling (Liu *et al*. 2014). Indeed, LPS inhibition of AMPK expression *in vivo* is not limited to adult tissue as a very recent paper described the same phenomenon in weaned piglets (Kang *et al*. 2014) and we ourselves find a rapid decrease in AMPKα1 and AMPKα2 mRNA expression in response to LPS in neonatal rats (Hagberg H, unpublished data).

In conclusion, we find that AMPK is rapidly activated in a mouse model of HI; replicating this injury *in vitro*, we show that it is mediated by CaMKKβ activation of AMPKα1 complexes. This aberrant AMPK activity during the insult period may act to exacerbate injury as conversely, AMPK inhibition reduces cell death. However, AMPK inhibition prior to injury sensitizes neurons to OGD stress. Further studies are required to determine whether bacterial inhibition of AMPK prior to HI results in a worse outcome in mouse models of HI and in neonatal HIE, and the molecular mechanisms are still to be examined in detail.

## Supporting information


**Figure S1.** Extent of injury in the Vannucci hypoxic–ischaemic mouse model.
**Figure S2.** Comparison of the mRNA expression of AMPKα1 and AMPKα2 in primary neurons Total mRNA was extracted from wild‐type primary neurons and analysed by one‐step qRT‐PCR for the expression of AMPK catalytic subunits.
