# Supplementary material for: A dual role for AMP‐activated protein kinase (AMPK) during neonatal hypoxic–ischaemic brain injury in mice
Source: J Neurochem. 2015 Feb 24;133(2):242–52. doi: 10.1111/jnc.13034 (PMC4855681; doi:10.1111/jnc.13034)
Supplement: Supplementary file 1 — Figure S1. Extent of injury in the Vannucci hypoxic–ischaemic mouse model. Figure S2. Comparison of the mRNA expression of AMPKα1 and AMPKα2 in primary neurons Total mRNA was extracted from wild‐type primary neurons and analysed by one‐step qRT‐PCR for the expression of AMPK catalytic subunits. [file JNC-133-242-s001.pdf]

Supplementary Figure 1

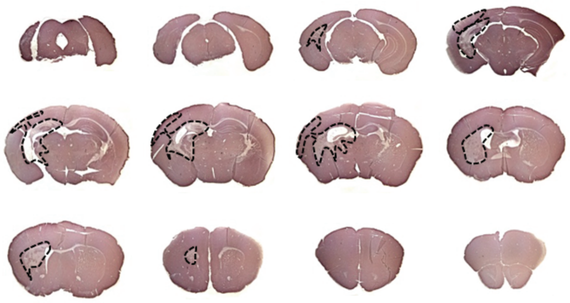

SFig 1. Extent of injury in the Vannucci hypoxic-ischaemic mouse model.

HI injury was induced in P9 mice and the extent of injury assessed by MAP2 staining of sequential coronal brain slices (posterior to anterior). Dotted lines define the area affected by the infarct.

Supplementary Figure 2

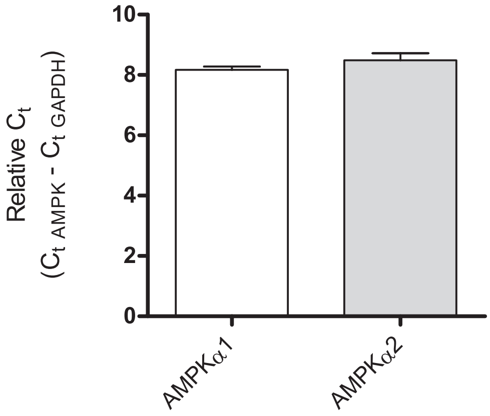

SFig 2. Comparison of the mRNA expression of AMPKα1 and AMPKα2 in primary neurons

Total mRNA was extracted from wild-type primary neurons and analysed by one-step qRT-PCR for the expression of AMPK catalytic subunits. Data were normalised relative to the expression of GAPDH. AMPKα1/α2 mRNA threshold detection (Ct) levels were not significantly different (N=4).
